# Effect of Combined Interval and Continuous Exercise Training on Gastric Emptying, Appetite, and Adaptive Responses in Men With Overweight and Obesity

**DOI:** 10.3389/fnut.2021.654902

**Published:** 2021-05-28

**Authors:** Katy M. Horner, Nuala M. Byrne, Neil A. King

**Affiliations:** ^1^School of Public Health, Physiotherapy and Sport Sciences, Institute for Sport and Health and Institute of Food and Health, University College Dublin, Dublin, Ireland; ^2^School of Exercise and Nutrition Sciences, Institute of Health and Biomedical Innovation, Queensland University of Technology, Brisbane, QLD, Australia; ^3^School of Health Sciences, College of Health and Medicine, University of Tasmania, Hobart, TAS, Australia; ^4^School of Exercise and Nutrition Sciences, Institute of Health and Biomedical Innovation, Queensland University of Technology, Brisbane, QLD, Australia

**Keywords:** appetite, energy intake, energy balance, cardiorespiratory fitness, compensatory responses, high intensity interval

## Abstract

**Background/Objectives:** Characterizing compensatory and adaptive responses to exercise assists in understanding changes in energy balance and health outcomes with exercise interventions. This study investigated the effects of a short-term exercise intervention (combining high intensity interval (HII) and continuous exercise) on (1) gastric emptying, appetite and energy intake; and (2) other adaptive responses including cardiorespiratory fitness, in inactive men with overweight/obesity.

**Methods:** Fifteen men (BMI: 29.7 ± 3.3 kg/m^−2^) completed a 4-wk supervised exercise intervention, consisting of 5 exercise sessions per week alternating between HII (30 s at 100% VO_2_max followed by 30 s recovery) and continuous (at 50% VO_2_max) training on a cycle ergometer, progressing from 30 to 45 min session duration. Gastric emptying (^13^C-octanoic acid breath test), appetite (visual analog scale), energy intake (*ad libitum* lunch meal), body composition (air displacement plethysmography), non-exercise activity (accelerometery) VO_2_max, blood pressure, and fasting concentrations of glucose, insulin, and ghrelin were measured before and after (≥48 h) the intervention.

**Results:** Gastric emptying, glucose, insulin and ghrelin were unchanged, but energy intake at the *ad libitum* lunch test meal significantly increased at post-intervention (+171 ± 116 kcal, *p* < 0.01). Body weight (−0.9 ± 1.1 kg), waist circumference (−2.3 ± 3.5 cm) and percent body fat (−0.9 ± 1.1%) were modestly reduced (*P* < 0.05). VO_2_max increased (+4.4 ± 2.1 ml.kg.min^−1^) by 13% and systolic (−6.2 ± 8.4 mmHg) and diastolic (−5.8 ± 2.2 mmHg) blood pressure were significantly reduced (*P* ≤ 0.01 for all).

**Conclusions:** Four weeks of exercise training did not alter gastric emptying, indicating gastric emptying may only adapt to a higher volume/longer duration of exercise or changes in other characteristics associated with regular exercise. The combination of HII and continuous exercise training had beneficial effects on body composition, cardiorespiratory fitness, and blood pressure and warrants further investigation in larger randomized controlled trials.

## Introduction

Exercise has many health benefits, including weight maintenance, and should be an effective weight loss strategy by increasing energy expenditure. However, the efficacy of exercise for weight loss is modest (1) and will depend on changes in other components of energy balance including energy intake and non-exercise activity (2). Although it is intuitive that exercise drives an increase in appetite and energy intake, the relationship between exercise and appetite is more complex. Evidence indicates that exercise improves the sensitivity of appetite control (3–8) and that exercise influences at least two processes of appetite control: both the drive to eat and the satiating efficiency of a meal (5). As the strength of these processes may determine whether individuals lose weight with exercise, understanding the effects of exercise on energy intake and the underlying mechanisms is vital.

Gastrointestinal peptides and gastric emptying (the rate at which food empties from the stomach) have an important integrative role in the short-term control of food intake. For example, a slower gastric emptying is associated with increased gastric distension, postprandial fullness and reduced energy intake at a subsequent test meal (9, 10). However, a slower gastric emptying also delays the interaction of nutrients with the intestine, blunting the release of satiety related gut peptides in individuals with obesity (11). The relative influence of intestinal and gastric signals on appetite may be influenced by factors such as the time interval between meals, characteristics of the individual or of the meal. Cross-sectional studies have shown gastric emptying is faster in active compared to inactive individuals (12–14), and is associated with activity energy expenditure (14). Faster gastric emptying has been proposed as a mechanism which may increase desire for food intake with chronic physical activity (12). We have also previously hypothesized that faster gastric emptying with chronic exercise could be one mechanism contributing to an overall increase in meal frequency and energy intake by reducing gastric distension and fullness, but improved ability to match daily energy intake to expenditure in active individuals through enhanced intestinal satiety signaling (15). However, such relationships have yet to be investigated. In addition, while cross-sectional studies can provide important information, they do not allow for a causal relationship between changes in gastric emptying with repeated exercise training to be determined.

Potential mechanisms contributing to changes in gastric emptying and energy intake include alterations in fasting ghrelin (16), blood glucose (17) and insulin sensitivity (18), which have been shown to change in response to exercise training (3, 19–23). Therefore, examining changes in these blood markers may provide further mechanistic insight into changes in appetite with exercise.

Compensatory responses in other components of energy balance, including activity outside of the prescribed exercise program are also important factors when considering exercise prescription for individuals with overweight and obesity (24–26). In addition to changes in energy intake, non-exercise activity may be influenced by exercise intensity and reduced to a greater extent as a compensatory response to high intensity exercise (27, 28). Thus, changes in non-exercise activity could potentially undermine beneficial effects of higher intensity exercise on total daily activity levels.

Combining high intensity interval (HII) exercise sessions with continuous lower intensity exercise sessions may serve to provide benefits for increasing both cardiorespiratory fitness, along with increasing the total amount of exercise–an important factor contributing to body weight and fat loss (29). Exercise programs (aimed at improving total daily activity, cardiorespiratory fitness, body composition, and adherence) should include a combination of low- and high-intensity exercise (30), and have been shown to result in substantial improvements in VO_2_max in trained and untrained individuals (31). However, to the best of our knowledge the effects of combining HII and moderate intensity continuous exercise on compensatory responses and other health-related outcomes have not been widely examined in individuals with overweight and obesity.

The present study was undertaken to investigate the effects of a 4-week exercise intervention (combining HII and continuous exercise) on (1) gastric emptying, appetite and energy intake; and (2) body composition, non-exercise activity, cardiorespiratory fitness and related health markers in inactive men with overweight and obesity.

## Materials and Methods

### Participants

Based on our previous work examining the reproducibility of gastric emptying in individuals with overweight and obesity without any intervention (32), a minimum of 15 participants was required to detect a mean difference of at least 10% for all gastric emptying parameters, with a power of 80% and α = 0.05. Participants were recruited in the university and local area. Inclusion criteria were: male, aged 18–60 years, BMI 25–40 kg.m^−2^, weight stable (± 4 kg over last 6 months), non-diabetic, no history of GI surgery or disorder, no medical conditions, and not taking any medication known to influence the outcome measures, willing to consume study test meals, not a heavy smoker (<10 per day) and inactive (participating in one structured exercise session or less per week and not engaged in strenuous work). All participants completed the Sports Medicine Australia pre-exercise screening questionnaire and those with any risk factors were required to present approval by their medical doctor prior to participation. Ethical approval was granted by Queensland University of Technology Research Ethics Committee, the study was conducted in accordance with the Declaration of Helsinki and all participants provided written informed consent prior to taking part. No incentive was provided.

### Design

Participants attended the laboratory on 2 separate test days (at least 48 h apart) in the week prior to the 4-week exercise intervention (baseline) and on 2 separate test days in the week following the exercise intervention (post-intervention) (at least 48 h after the last exercise session to avoid any acute effects of exercise). At one testing session, fasting blood samples, body composition and VO_2_max were measured. At the second test session, gastric emptying, subjective appetite sensations and *ad libitum* lunch energy intake were assessed. The order of testing sessions was the same for all participants. On both occasions, participants attended the laboratory after a 12 h overnight fast, and having avoided alcohol and strenuous exercise for 24 h. One glass of water was allowed upon waking. Participants were instructed to repeat these procedures prior to the post-test. There was no dietary intervention, similar to others assessing the impact of exercise without dietary intervention (23, 33–35).

#### Exercise Intervention

The exercise intervention consisted of five exercise sessions per week for 4 weeks. All sessions were supervised and involved indoor cycling on a cycle ergometer (Monark 884E Ergomedic Sprint Bike, Monark Exercise AB, Vansbro, Sweden). Exercise sessions alternated between continuous cycling and HII exercise, with participants prescribed ten of each type over the course of the 4 weeks. The continuous exercise sessions involved cycling at a constant workload equivalent to 50% VO_2_max for the duration of the session. HII sessions consisted of 30 s cycling at 100% VO_2_max followed by 30 s recovery (unloaded cycling or static recovery) each minute for the duration of the session. Thus, an identical relative workload and time duration was prescribed.

Exercise duration progressed by 5 min/week from 30 min in week 1 to 45 min in week 4. Each session started with a 5 min warm up of unloaded cycling and finished with a cool down. Participants wore a heart rate monitor (Polar Electro Oy, Kempele, Finland) during each exercise session. HR and RPE using the Borg Scale (36) were recorded every 5 min. In HII sessions, recordings were taken immediately at the end of a HII bout. Workloads were prescribed based on each participant's baseline VO_2_max test using individual regression equations for each subject. Percent VO_2_max data calculated during the last 30 s of each stage of the test was plotted against stage workload and 50% and 100% VO_2_max were used to calculate the corresponding prescribed workloads.

#### Anthropometry and Body Composition

Height was measured without shoes to the nearest 0.5 cm and weight to the nearest 0.01 kg. Waist and hip circumferences were taken and body composition was measured using air displacement plethysmography (Bodpod, Concord, CA).

#### Maximal Oxygen Consumption (VO_2_ Max)

VO_2_ max was assessed using a TrueMax 2400 Metabolic Cart (ParvoMedics Inc, USA). All tests were conducted on the same cycle ergometer (Monark Bike 839E, Monark Exercise AB, Sweden) and consisted of 2 phases [similar to Wood et al. (37)]. Phase 1 consisted of a graded exercise test performed to volitional exhaustion and phase 2 consisted of a verification test. Participants were instructed to maintain cycling cadence at 70 rpm. Participants performed a 2-min warm up at the start of the graded test. Subsequently, workload was increased each minute by either 21 or 28 W (determined prior to the test based on the participant's predicted VO_2_max). Following phase 1, the participant was given a 5-min rest and a small glass of water. Participants then resumed cycling at the workload of the third last 1-min stage of the preceding maximal continuous incremental test for phase 2 (the verification test) (37). The workload was increased each minute until volitional exhaustion. This two-phase test was used as it has been suggested that a verification or “booster” test may provide a time-efficient means of verifying whether a VO_2_peak is indicative of a true maximal VO_2_ (38).

The continuous incremental exercise test (phase 1) was deemed to be a valid maximal test on the basis of achievement of at least three of the following criteria during the final 30 s of the last completed stage (37): Increase in VO_2_ <50% of that expected for the change in mechanical work, heart rate (HR) within +/– 11 bpm of age-predicted maximum, calculated as 220 –age, respiratory exchange ratio (RER) ≥ 1.15, RPE ≥ 18. Ventilatory threshold was calculated using the combined approach (39).

#### Blood Pressure

Systolic and diastolic blood pressure were assessed using an Omron IA1B blood pressure monitor (Omron Healthcare Singapore PTE Ltd, Singapore) in a seated position. Measurements were taken in duplicate following 10 min of sitting to ensure the participant was rested and relaxed.

#### Blood Samples

Fasting samples were collected by venepuncture into potassium oxylate, serum and EDTA tubes containing aprotinin and DPP-IV inhibitors. Potassium oxylate and EDTA tubes were immediately centrifuged (refrigerated at 2,000 g for 10 min) and the serum tube was allowed to stand for 30 min before centrifugation. Samples were immediately aliquoted, placed in liquid nitrogen and stored at −80 degrees until analysis. Plasma glucose was measured colourimetrically using standard laboratory techniques, insulin by chemiluminescent immunoassay and total ghrelin using an established RIA. All analyses were conducted in duplicate, and mean values are reported. Intra-sample CV's were 0.7 ± 1.1% for glucose, 2.1 ± 1.3% for insulin and 6.5 ± 5.2% for ghrelin. Insulin resistance by homeostasis model (HOMA-IR) was calculated according to Matthews et al. (40): HOMA-IR = fasting glucose × fasting insulin/22.5.

#### Non-exercise Activity

Non-exercise activity (i.e., activity outside of the prescribed exercise) was monitored using a tri-axial GT3X accelerometer (Actigraph, Fort Walton Beach, FL, USA). Participants were provided with the accelerometer to wear for 7 days prior to the intervention and again in week 4 of the intervention, a duration estimated to result in 90% reliability (41). The accelerometer was attached to an elastic belt and worn on the waist, in line with the right hip. Data were processed using ActiLife software (version 6.4.5). VM3 counts were summed over 60 s epochs and levels of activity were defined as counts per minute using cut point values according to validated recommendations (42). Data were checked for spurious values (counts per minute of >15,000). A non-wear period was defined as at least 90 min of consecutive zero counts without interruption (43). Wear time exceeding 600 min was considered a valid day (44) and a valid dataset was considered a combination of at least 3 week days and 1 weekend day (45). Data during prescribed exercise times were excluded from analysis. Mean minutes per day of time spent in moderate and vigorous (combining vigorous and very vigorous) activity were calculated. Activity count data were converted to activity energy expenditure (AEE) using the “Freedson VM3 combination (11)” option in Actilife software (version 6.4.5). Accelerometery data were compared between pre- and post- exercise intervention. Data were also compared in participants who had a complete 24 h dataset following a single continuous and HII exercise session in week 4 to examine whether subsequent 24 h AEE was impacted by the type of training session.

#### Energy Compensation

To estimate energy expenditure from the prescribed exercise, individual energy expenditure regression equations were developed for each participant using the heart rate and energy expenditure values recorded during the last 30 s of each stage of the VO_2_max test, similar to previous work (46–48). Heart rates recorded during the prescribed exercise sessions were then inserted into the individual regression equations to predict energy expenditure. Net energy cost of exercise was calculated by subtracting resting energy expenditure from energy expenditure during prescribed exercise. Resting energy expenditure was measured over 30 min at baseline by indirect calorimetry using an identical procedure to previous work (14).

Energy compensation was calculated following Riou et al. (46, 49) based on the total estimated energy expended during prescribed exercise (EE), and changes in fat (FM) and fat free mass (FFM) observed using energy equivalents for fat mass and fat free mass previously described (50) as follows:

$$\begin{array}{l} \text{Energy Compensation }\left(\%\right)\text{= }\frac{\text{100}}{\text{EE }(\text{kcal})}\;\\ \;\times \;[\left(\text{FM }\left(\text{kg}\right)\;\times \text{ 9},\text{500 kcal}\right)\\ \text{+ }\left(\Delta \text{FFM }\left(\text{kg}\right)\;\times \text{ 1},\text{020 kcal}\right)]\text{+100} \end{array}$$

Using this method compensation of 0% indicates changes in body composition following the intervention matched expected changes based on exercise EE. A positive value indicates changes in body energy stores are less than expected, with a value of 100% indicating body composition remained the same. In contrast, a negative value indicates body energy stores are reduced beyond what would be expected based on exercise EE (46, 49).

#### Gastric Emptying Test Day Measurements

##### Gastric Emptying

Gastric emptying parameters were calculated using the ^13^C-octanoic acid breath test (51), using an identical procedure to that described in detail previously (32). In brief, the egg yolk of a standardized pancake breakfast meal [400 kcal; 15 g (15%) PRO, 17 g (37%) Fat, 48 g (48%) CHO)] was labeled with 100 mg ^13^C-octanoic acid (Cambridge Isotope Laboratories, Andover, USA). Participants consumed the meal together with 250 ml of water within 10 min. Breath samples were collected in 10 ml glass Exetainer tubes (Labco, Buckinghamshire, UK) prior to the breakfast, immediately after, and subsequently at 15 min intervals for 5 h after breakfast. Participants remained in sedentary activities throughout. No food or drinks were provided to participants during this time. ^13^C enrichment of breath samples was measured by isotope ratio mass spectrometry (Hydra 20–20) and compared to a reference gas (5% CO_2_, 75% N_2_, 20% O_2_ calibrated with a standard of ^13^CO2). Data were analyzed according to Ghoos et al. (51). The conventional uncorrected time based parameters (t_lag_ and t_1/2_) proposed by Ghoos et al. (51) and the parameters latency time (t_lat_) and ascension time (t_asc_) proposed by Schommartz et al. (52) were calculated.

##### Subjective Appetite Sensations

Subjective appetite sensations were measured throughout the test day using an electronic appetite rating system. Participants were asked to rate feelings of hunger, fullness and desire to eat on 100 mm visual analog scales, using an identical protocol to previous work (53). The satiety quotient (SQ) (54) was calculated for each sensation at breakfast and palatability ratings of both breakfast and lunch meals were assessed immediately post-meal using 100mm visual analog scales.

##### AD Libitum Energy Intake

At the end of the gastric emptying test, participants were provided with an *ad libitum* pasta lunch meal identical to that described previously (53) (47% CHO, 35% FAT, and 18% PRO, and an energy content of 1.8 kcal/g) and water and instructed to consume as much as they wished until comfortably full. The amount (g) of food consumed was determined by weighing the meal before and after consumption and energy intake (kcal) calculated.

### Statistical and Data Analysis

Data are presented as mean values and standard deviations (SD). Changes from pre- to post- exercise intervention were assessed using paired sample *t*-tests. Unless otherwise stated Pearson correlations were used to determine relationships between changes in key variables. Spearman correlations were used for non-parametric data. Area under the curve for appetite ratings was calculated using the trapezoidal rule. Following a similar approach to King et al. (55), in order to provide insight into individual variability in responses that may be attributed to the exercise intervention, normal day-to-day variability in the key outcome measures is considered by graphically presenting the findings in relation to our previous work examining the reproducibility of gastric emptying (32) and energy intake (53) in a similar population of men with overweight/obesity without intervention. Statistical analysis was carried out using PASW Statistics 18.0 (SPSS Inc., Chicago, IL) and statistical significance accepted at *p* < 0.05.

## Results

Eighteen men met the inclusion criteria and three withdrew during the intervention, resulting in fifteen males completing the study. Three participants did not complete the 4 week exercise intervention–two due to time commitments and personal circumstances and one participant was excluded due to insufficient attendance at exercise sessions. Results are presented for 15 men (BMI: 29.7 ± 3.3, Age: 31.1 ± 8.4 yr) who completed all parts of the study.

### Exercise Intervention Characteristics and Energy Compensation

Participants completed 96 (3.9)% of the prescribed number of exercise sessions, with all participants completing a minimum of 90% (18 of 20) of the exercise sessions. Mean RPE decreased by 1.7 units during continuous and 2.2 units during HII exercise respectively, when compared over the first 30 min from week 1 to week 4 (*p* < 0.001). Mean total time spent in prescribed exercise was 705 ± 43 min. Mean total energy expended in prescribed exercise calculated over the 4 week intervention was 7,803 ± 1,587 kcal. Mean energy compensation was −41 ± 136%, indicating that on average participant's energy stores were reduced to a greater extent than would have been expected based on exercise energy expenditure. However, individual values ranged from −315 to 214% indicating inter-individual differences in responses were highly variable. In total, five had positive values (range 5–214%) indicating energy compensation occurred and ten negative values (range −20 to −315%) indicating greater reductions in energy stores than expected.

### Anthropometry, Body Composition, Blood Pressure, and Fitness

The small reductions in weight, BMI, body fat, and waist circumference at the end of the intervention were statistically significant (Table 1). Weight change ranged from −2.4 kg loss to + 0.8 kg gain, and as a percentage of initial body weight from −3.0% loss to 0.9% gain. Systolic and diastolic blood pressure were significantly reduced and there was a significant increase in VO_2_max (mean 12.8% increase in ml.kg.min^−^^1^; mean 11.6% increase in L.min^−1^) (Table 1). Four participants did not meet the criteria for VO_2_max at pre- and post- test, however the verification test indicated that they could not complete any additional stages. Mean RER (Pre: 1.15 ± 0.04, Post: 1.13 ± 0.06) and HR_max_ (Table 1) during the final 30 s of the last completed stage did not differ significantly between pre- and post-test.

**Table 1:** Participant anthropometric, body composition, cardiorespiratory fitness, and blood pressure characteristics pre- and post- 4-week exercise intervention (*n* = 15).

	**Pre**	**Post**	***P*-value**
Weight (kg)	95.6 ± 13.0	94.7 ± 13.0	**<0.01**
BMI (kg.m^−2^)	29.7 ± 3.3	29.3 ± 3.2	**<0.001**
**Body composition**		
Body fat (%)	30.0 ± 6.8	29.0 ± 6.7	**0.01**
FFM (kg)	66.4 ± 7.1	66.7 ± 6.8	0.50
Waist (cm)	97.1 ± 9.6	94.9 ± 8.7	**0.03**
Hip (cm)	107.8 ± 7.3	107.2 ± 7.2	0.15
**Fitness**		
VO_2_max (ml.kg.min^−1^)	34.3 ± 5.9	38.7 ± 5.9	**<0.001**
VO_2_max (L.min^−1^)	3.25 ± 0.57	3.63 ± 0.52	**<0.001**
HR max (bpm)	183 ± 13	182 ± 8	0.51
Workload max (Watts)	270 ± 51	308 ± 48	**<0.001**
Ventilatory threshold (Watts)^a^	136 ± 35	187 ± 33	**<0.001**
Ventilatory threshold (%VO_2_max)^a^	53 ± 11	61 ± 5	**0.001**
**Blood pressure**		
Systolic (mmHg)	122 ± 8	116 ± 9	**0.01**
Diastolic (mmHg)	79 ± 7	74 ± 9	**<0.01**

*Data are means ± SD*.

*BMI, body mass index; FFM, fat free mass; VO_2_max, maximum oxygen uptake; HR, heart rate*.

a *Combined VT calculations are reported for n = 14 as the time of VT for one participant occured at <4 min, therefore the data was rejected as per (39). Bold highlights statistically significant values*.

### Gastric Emptying and Blood Parameters

Gastric emptying, fasting ghrelin, glucose, insulin and HOMA-IR did not significantly differ between pre- and post-exercise intervention (Table 2).

**Table 2:** Gastric emptying time based parameters, fasting ghrelin, glucose, insulin, and HOMA-IR Pre and Post 4 week exercise intervention (*n* = 15).

	**Pre**	**Post**	***P*-value**
GE t_lag_ (min)	111 ± 17	110 ± 18	0.71
GE t_1/2_ (min)	175 ± 22	179 ± 25	0.25
GE t_lat_ (min)	37 ± 9	35 ± 8	0.09
GE t_asc_ (min)	137 ± 17	144 ± 21	0.10
Fasting ghrelin (ng/L)	805.4 ± 337.6	760.8 ± 331.0	0.12
Fasting glucose (mmol/L)	5.49 ± 0.30	5.44 ± 0.22	0.39
Fasting insulin (mIU/L)	9.40 ± 4.66	8.70 ± 4.18	0.19
HOMA-IR	2.30 ± 1.16	2.11 ± 1.05	0.20

*Data are means ± SD*.

*GE, gastric emptying; t_1/2_, half time; t_lag_, lag time; t_asc_, ascension time; t_lat_, latency time*.

**Figure 1:**
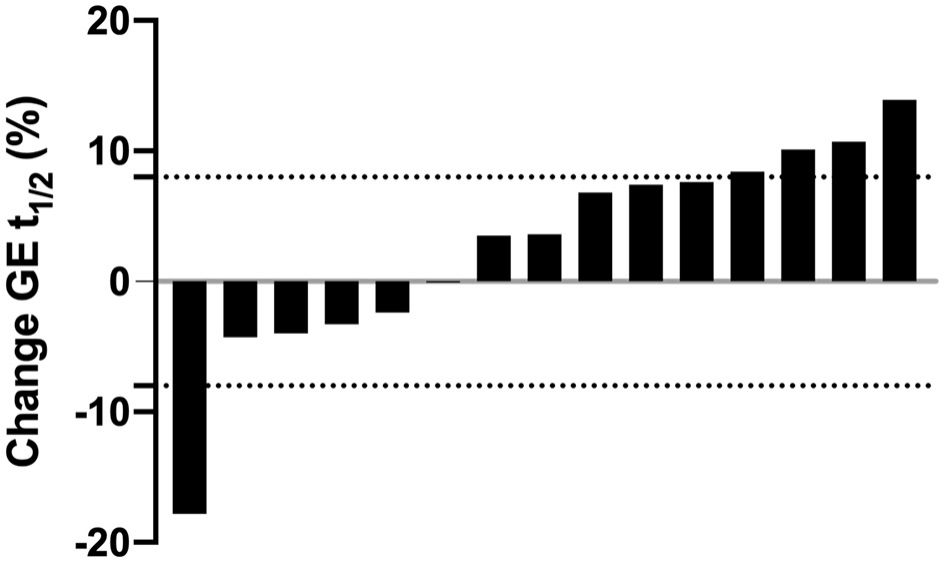
Individual changes in gastric emptying half time (GE t_1/2_) expressed as percentage change from baseline after the 4-week exercise intervention. Each bar represents an individual participant (*n* = 15). Values above zero indicate a longer (i.e., slower), values below zero indicate a shorter (i.e., faster) GE t_1/2_ after the intervention. Dashed horizontal lines represent zones of natural variation in GE t_1/2_ (± 8%) based on our previous work (32).

Despite no mean changes, there was variability in changes in these outcome measures. Six individuals had a faster t_1/2_ at post-test, ranging from 0.1 to 17.8% (0.2 to 32.0 min) faster, and nine individuals had a slower t_1/2_ at post-test, ranging from 3.5 to 13.9% (5.5 to 25.0) min slower. However, most changes were within the intra-individual CV of 8% identified in our previous work (32) (Figure 1). Comparing the results to the natural variation previously documented, the changes in GE t_1/2_ of 66% (*n* = 10) of participants fell within this normal range.

### Appetite Ratings and *ad libitum* Test Meal EI

Subjective appetite ratings are shown in Supplementary Figure 1 and did not differ significantly between pre- and post- exercise intervention for fasting, mean 5 h, 5 h AUC and breakfast satiety quotient (*p* > 0.14 for all). In addition, there were no significant differences between pre- and post-intervention for palatability ratings of the breakfast and lunch meals (*p* > 0.09 for all).

EI at the *ad libitum* lunch test meal was significantly higher following the exercise intervention (Pre: 712 ± 173 kcal, Post: 883 ± 159 kcal, *p* < 0.001), with a mean 27% increase from baseline. Variability in individual changes in energy intake are shown in Supplementary Figure 2, illustrating most changes were outside the intra-individual CV of 12% identified in our previous work (53). Comparing the results to the natural variation previously documented, the changes in energy intake of 73% (*n* = 11) of participants fell outside the normal range.

### Non-exercise Activity

Due to two invalid accelerometery data sets, physical activity data is reported for *n* = 13. Wear duration was significantly less at post-intervention (pre: 893 ± 73 min and post 826 ± 72 min, *p* = 0.03), due to the time in prescribed exercise being excluded from calculations. Non-exercise activity did not significantly differ between pre-intervention and week 4 (Table 3). When controlling for the difference in wear time, there was a significant interaction effect for AEE (*p* = 0.04) and steps per day (*p* = 0.01) but not for time spent in moderate or vigorous activity. Including EE from the prescribed exercise sessions, there was a significant increase in average daily AEE of +303 ± 162 kcal over week 4 of the intervention compared to pre-intervention (*p* = 0.007).

**Table 3:** Mean physical activity characteristics at baseline and during week 4 (excluding prescribed exercise) of the 4 week exercise intervention (*n* = 13).

	**Pre**	**Week 4**	***P*-value**
**Physical activity**		
Steps per day	*6, 714*±*2, 082*	*6, 718*±*2, 399*	0.99
AEE (kcal/day)	568 ± 196	579 ± 227	0.16
**Time in activity**		
Vigorous (min/day)	5 ± 4	5 ± 5	0.97
Moderate (min/day)	42 ± 18	42 ± 22	0.97

*Data are means ± SD*.

*AEE, activity energy expenditure estimated from accelerometery*.

Data was available for a complete 24 h period for *n* = 11 participants after both a HII and continuous exercise session in week 4. Non-exercise activity did not differ in the 24 h after a single continuous exercise session compared to after a HII session in week 4 (Supplementary Table 1; Supplementary Figure 3). Wear duration did not differ between conditions (*p* = 0.14).

### Relationships Among Energy Compensation, Anthropometric, Body Composition, Gastric Emptying, Blood Markers, Physical Activity, Appetite, and *ad libitum* Energy Intake Changes

Energy compensation was inversely associated with change in AEE (including prescribed exercise) (*r* = −0.61, *p* = 0.03), indicating a greater increase in AEE in week 4 was associated with less energy compensation. Energy compensation was also associated with change in body fat [percent (*r* = 0.84, *p* < 0.001) and kg (*r* = 0.97, *p* < 0.001)], change in fasting insulin (*r* = 0.56, *p* = 0.03) and change in HOMA-IR (*r* = 0.51, *p* = 0.05), indicating lower energy compensation was associated with a greater reduction in body fat, insulin and HOMA-IR. However, energy compensation was not associated with changes in FFM (kg, *r* = −0.43, *p* = 0.11), gastric emptying or any other variables.

There was a trend toward a negative correlation between change in gastric emptying t_asc_ with average daily AEE (including AEE in the prescribed exercise sessions) (*r* = −0.53, *p* = 0.06). Change in t_asc_ was also negatively correlated with change in AEE outside of the prescribed exercise (*r* = −0.67, *p* = 0.01) and similar negative correlations were found between change in t_asc_ and changes in steps per day (*r* = −0.65, *p* = 0.02) and mean time in vigorous activity per day (*r* = −0.64, *p* = 0.02) outside of prescribed exercise. These findings indicate a greater increase in activity was associated with shorter (i.e., faster) gastric emptying time following the intervention. However, changes in gastric emptying were not correlated with changes in anthropometric, body composition, blood markers, VO_2_max, appetite (5 h mean or AUC) or *ad libitum* test meal energy intake variables. Change in AEE (including prescribed exercise) was also inversely associated with change in body fat (kg) (*r* = −0.58, *p* = 0.04), indicating a greater increase in AEE in week 4 was associated with less energy compensation and a greater reduction in body fat at post-intervention. Change in AEE (excluding prescribed exercise) was not correlated with time spent in prescribed exercise (Spearman rho = 0.02, *p* = 0.95), indicating overall exercise participation was not associated with change in AEE outside of the intervention.

A decrease in fasting insulin and HOMA-IR from pre-to post-intervention was associated with a decrease in body fat (insulin: *r* = 0.69, *p* = 0.004; HOMA-IR: *r* = 0.67, *p* = 0.006). Fasting ghrelin was not associated with changes in other variables. Change in *ad libitum* test meal energy intake was associated with change in percentage body fat (*r* = 0.52, *p* = 0.048) but not with other variables, indicating an increase in energy intake was associated with a lesser reduction in body fat following the intervention.

## Discussion

The present findings demonstrate that in response to a 4-wk exercise intervention combining HII and continuous exercise (1) gastric emptying, glucose, insulin, ghrelin, appetite ratings and non-exercise activity are unaltered despite an increase in *ad libitum* test meal energy intake; and (2) body composition, cardiorespiratory fitness and blood pressure are improved, in men with overweight and obesity. Compliance was high (≥90% completion of all sessions), the intensity and frequency of sessions were high, each session was supervised in the laboratory and the intervention resulted in a significant improvement in cardiorespiratory fitness. It can therefore be reasonably concluded from the present findings that in the short to medium term (4 weeks), in the absence of acute exercise effects gastric emptying is unaltered in response to exercise training in men with overweight and obesity.

In contrast, cross sectional studies have shown faster gastric emptying in active compared to inactive men (13). In a previous study demonstrating faster gastric emptying in marathon runners, the runners were training for a mean 4.9 years (13) and in our previous work, habitual exercisers were defined as individuals engaged in 4 or more exercise sessions per week for a minimum of 6 months (14). Therefore, gut adaptations (i.e., faster gastric emptying) in response to regular exercise may only occur after a much longer period of time than the 4-week intervention in the present study. Interestingly, the only significant correlates of changes in gastric emptying parameters over time were changes in activity assessed by accelerometer outside of the prescribed exercise sessions. A greater increase in activity between baseline and week 4 was associated with a faster gastric emptying time. In addition, there was a trend toward a greater increase in average daily AEE including time in prescribed exercise being associated with a shorter (i.e., faster) gastric emptying time. These findings are consistent with our previous cross-sectional evidence showing significant associations between greater activity, AEE and faster gastric emptying (14). In the present study, activity was restricted in the 48 h prior to gastric emptying testing, which could be one factor explaining why gastric emptying and appetite were unchanged overall. Further studies are warranted to investigate this and the temporal pattern of changes in gastric emptying and associations with appetite and daily EI with longer term interventions and more substantial weight loss.

The rationale for the 4 week duration of intervention was to investigate the effects of the exercise intervention on gastric emptying and compensatory responses before substantial changes in body composition were likely to occur and impact responses. Some evidence shows change in VO_2_max is associated with energy compensation (47, 48). In contrast, the exercise intervention in the present study demonstrated on average a significant improvement in VO_2_max without energy compensation or changes in gastric emptying. Week 4 exercise intervention responses may also be useful in determining longer term effects of exercise on weight loss and compensatory responses in individuals with overweight or obesity (56). However, a limitation of the current study is that the sample size limited further understanding of individual responses. Only 5 participants had positive energy compensation values indicating a degree of compensation for exercise energy expenditure. Further studies with a larger numbers of participants would assist to better understand whether alterations in gastric emptying may be a compensatory mechanism occurring in individuals identified as “compensators” compared to “non-compensators” with exercise intervention.

The 4-week exercise intervention also had no significant effect on fasting glucose, insulin, HOMA-IR or total ghrelin, and could provide further explanation behind why no changes in gastric emptying were observed. Excluding the effects of acute exercise may similarly be one explanation for the lack of changes in fasting insulin and HOMA-IR. Although, it has long been established that a single bout of exercise improves glucose metabolism acutely (57), the benefits of exercise appear to diminish within 48 to 72 h of the last exercise session (23, 58). The timing of the post-testing (≥ 48 h after last exercise) in the present study was selected to allow the effects of short-term exercise training in the absence of acute exercise effects to be established, and to allow comparison with previous studies examining the effects of exercise training on gut peptides (3, 20, 21, 59).

In addition, although changes in body composition were significant, the reductions in body fat were modest (mean ~1% change) and it is likely that more substantial changes in body fat may be required to improve insulin sensitivity in response to chronic exercise. Indeed, we observed that changes in insulin and HOMA-IR were strongly associated with reductions in body fat. These findings support the contention that the chronic impact of exercise training on insulin may be mediated by reduced adiposity (57). The present findings are also consistent with evidence that fasting ghrelin levels appear to be unaffected by exercise training in the absence of significant concurrent weight loss (60).

Subjective appetite sensations were similarly unchanged after the 4-week intervention, consistent with some previous studies showing no change in appetite ratings following both 7 and 14 days of exercise in lean men and women (61, 62). However, in response to longer-term interventions, changes in subjective appetite ratings have been documented (5, 63) suggesting appetite ratings may only respond to longer duration interventions.

Despite no significant changes in appetite ratings, *ad libitum* energy intake at the lunch test meal increased (mean 171 kcal higher (27% increase), which equated to ~32% of the average energy expended in a prescribed exercise session in week 4). Although AEE was not associated with lunch test meal energy intake in the present study, AEE has been previously identified as an independent predictor of mean daily energy intake and to have a small contribution to the drive to eat (64, 65). Others have shown partial compensation in energy intake of ~30% for exercise induced EE following 14 days of high exercise levels in lean men (62). Although, in the latter study daily food intake was measured, the findings of a significant increase in energy intake at the *ad libitum* lunch in the present study could be indicative of a partial compensation in energy intake. Indeed, an increase in test meal energy intake was associated with a lesser reduction in body fat in response to the intervention. As gastric emptying was unchanged, other factors such as changes in leptin (66), cognitive factors such as attitudes and beliefs (e.g., exercise makes you hungry), a desire for self-reward after exercise and misjudgements about the amount of energy expended relative to energy intake (67, 68) could have contributed to the change in *ad libitum* energy intake at the lunch test meal. It is therefore important to address a range of factors which may contribute to compensatory increases in energy intake and thus impede weight/fat loss when individuals commence an exercise program for weight management.

A second major aim of the present study was to examine the effects of the intervention on cardiorespiratory fitness and other adaptations to exercise. We observed a mean 13% increase in VO_2_max [(+4.4 ml.kg.min^−1^); 12% in L.min^−1^ (+0.38 L.min^−1^)]. Previous studies in men with overweight and obesity involving HII only interventions have reported a mean 8% (+0.25 L.min^−1^) increase in VO_2_peak following 2 weeks (23), 7% (+1.9 ml.kg.min^−1^) increase following 4 weeks (34) and 2.8% (+0.84 ml.kg.min^−1^) increase following 6 weeks (69) of HII training 3 days per week. Others (70) have reported a 13% (+0.4 L.min^−1^) increase in VO_2_max in overweight males following 12 weeks of HII training three times per week. Interestingly, the total prescribed exercise time in that study [720 min (70)] was similar to the present study (750 min). However, compared to the latter study we observed a similar improvement in VO_2_max following just 4 weeks of training and with less total training sessions (20 vs. 36) and less HII sessions (10 vs. 36).

Limited research has examined the effects of combining HII and continuous exercise interventions in healthy individuals with overweight and obesity. However, this type of training has been shown to be well-tolerated in a small study of individuals with overweight and obesity (71). Moreover, in individuals with Type 2 diabetes, Mourier et al. (72) examined the effects of combined continuous (2 days per week) and HII exercise (once per week) for 8 weeks, and observed a substantial improvement in VO_2_peak [41% (+9.4 ml.kg.min^−1^) increase] and reductions in adiposity. The addition of continuous to HII exercise sessions, thus increasing the total exercise dose and energy expenditure, is one possible explanation for the improvements in VO_2_max and body composition. Molecular mechanisms such as an increase in PGC1-α with combined interval and continuous exercise (73) could also potentially contribute to the significant changes in VO_2_max observed. Taken together, the current findings demonstrate that a short-term intervention combining HII and continuous exercise has beneficial effects on cardiorespiratory fitness, body composition and blood pressure.

A final objective was to compare the effects of the intervention on non-exercise activity. We found no change in non-exercise activity between baseline and the final week of the exercise intervention. Although other studies using doubly labeled water to quantity total EE have shown reductions in non-exercise activity in women with overweight and obesity (24, 46), the present findings are consistent with a systematic analysis (74) and some longer term studies in adults with overweight and obesity (63, 75). The present data also suggest the prescription of both HII and continuous moderate intensity exercise are effective for increasing total daily activity levels during an exercise intervention in men with overweight and obesity.

We acknowledge that the sample size limits the ability to generalize the findings, although it is similar to other studies in this area (3, 23). The study was undertaken in males to minimize confounding effects of menstrual cycle on key outcomes. In some studies examining changes in non-exercise activity and energy intake to exercise training in females, compensation has been demonstrated (24, 46, 63), therefore results in females may differ and further studies in females are warranted. Exercise EE was not directly measured during all exercise sessions, as indirect calorimetry during each training session was not feasible. In addition a constant load exercise test to estimate EE was not undertaken. Test meal intake as assessed in the present study provides an objective measurement but does not necessarily reflect daily changes, which represents a limitation of the current study. For example, Myers et al. (63) demonstrated an increase in daily energy intake but not lunch or dinner test meal intake following exercise intervention in women with overweight and obesity. It should also be noted that there was no dietary intervention, similar to others assessing the impact of exercise without dietary intervention (23, 33–35) and there was similarly no control group (3, 23, 63). However, the study was well-powered to detect significant changes in the primary outcome measure based on our previous work examining the reproducibility of gastric emptying in this population without intervention (32), all exercise sessions were supervised in the laboratory, exercise compliance was high (96%) and the “booster” VO_2_max test provided verification VO_2_peak was indicative of a true maximal VO_2_.

Adherence and perceived difficulty of exercise are important factors for sustaining long-term participation in physical activity. The exercise intervention used represented a considerable change in lifestyle for inactive individuals. Given that others have shown that health benefits can be achieved with less intense HII protocols (23), more research is needed to identify the optimal interval protocol for improving health outcomes. Although achievable, participants reported that the sessions, in particular the HII sessions, were more difficult at the beginning of the intervention. However, anecdotally some participants preferred HII and others continuous exercise. Others have shown untrained adults reported greater enjoyment after a single bout of HII as compared to continuous exercise (76). Moreover, HII exercise has been reported to be perceived as “more motivating” and continuous exercise “quite boring” (77). Combining both types of training may therefore further serve to provide variety.

In conclusion, firstly, 4 weeks of exercise training did not alter gastric emptying, glucose, insulin, ghrelin or subjective appetite ratings in the present study of inactive men with overweight and obesity. In the absence of acute exercise effects, these measures may only adapt to a greater volume of exercise or changes in other characteristics associated with regular exercise. Further longer term interventions are needed to characterize the temporal pattern of changes in gastric emptying with regular exercise and the underlying mechanisms. Secondly, the intervention combining continuous and HII exercise had beneficial effects on cardiorespiratory fitness, body composition and blood pressure and appeared not to alter non-exercise activity. Randomized controlled trials of larger sample sizes directly examining the efficacy of a combination of continuous and HII exercise compared to continuous and HII exercise only interventions would be of interest for future investigations to determine whether a combination intervention is more effective than either intervention alone.

## Data Availability Statement

The raw data supporting the conclusions of this article will be made available by the authors, without undue reservation.

## Ethics Statement

The studies involving human participants were reviewed and approved by Queensland University of Technology Research Ethics Committee. The patients/participants provided their written informed consent to participate in this study.

## Author Contributions

KH, NB, and NK: conceptualisation, writing, review, and editing. KH: data collection, formal analysis, and writing—original draft. All authors contributed to the article and approved the submitted version.

## Conflict of Interest

The authors declare that the research was conducted in the absence of any commercial or financial relationships that could be construed as a potential conflict of interest.

## References

[1] O'Donoghue G Blake C Cunningham C Lennon O Perrotta C. What exercise prescription is optimal to improve body composition and cardiorespiratory fitness in adults living with obesity? A network meta-analysis. Obes Rev. (2021) 22:e13137. 10.1111/obr.13137 32896055 PMC7900983

[2] King N Horner K Hills A Byrne N Wood R Bryant E. Exercise, appetite and weight management: understanding the compensatory responses in eating behaviour and how they contribute to variability in exercise-induced weight loss. Br J Sports Med. (2012) 46:315–22. 10.1136/bjsm.2010.082495 21596715

[3] Martins C Kulseng B King NA Holst JJ Blundell JE. The effects of exercise-induced weight loss on appetite-related peptides and motivation to eat. J Clin Endocrinol Metab. (2010) 95:1609–16. 10.1210/jc.2009-2082 20150577

[4] Van Walleghen EL Orr JS Gentile CL Davy KP Davy BM. Habitual physical activity differentially affects acute and short-term energy intake regulation in young and older adults. Int J Obes. (2007) 31:1277–85. 10.1038/sj.ijo.0803579 17342074

[5] King NA Caudwell PP Hopkins M Stubbs JR Näslund E Blundell JE. Dual-process action of exercise on appetite control: increase in orexigenic drive but improvement in meal-induced satiety. Am J Clin Nutr. (2009) 90:921–7. 10.3945/ajcn.2009.27706 19675105

[6] Beaulieu K Hopkins M Blundell J Finlayson G. Does habitual physical activity increase the sensitivity of the appetite control system? A systematic review. Sports Med. (2016) 46:1897–919. 10.1007/s40279-016-0518-9 27002623 PMC5097075

[7] Beaulieu K Hopkins M Long C Blundell J Finlayson G. High habitual physical activity improves acute energy compensation in nonobese adults. Med Sci Sports Exerc. (2017) 49:2268–75. 10.1249/MSS.0000000000001368 28692632

[8] Beaulieu K Hopkins M Blundell J Finlayson G. Homeostatic and non-homeostatic appetite control along the spectrum of physical activity levels: an updated perspective. Physiol Behav. (2018) 192:23–9. 10.1016/j.physbeh.2017.12.032 29289613

[9] Santangelo A Peracchi M Conte D Fraquelli M Porrini M. Physical state of meal affects gastric emptying, cholecystokinin release and satiety. Br J Nutr. (1998) 80:521–7. 10.1017/S0007114598001615 10211050

[10] Halawi H Camilleri M Acosta A Vazquez-Roque M Oduyebo I Burton D. Relationship of gastric emptying or accommodation with satiation, satiety, and postprandial symptoms in health. Am J Physiol Gastro Liver Physiol. (2017) 313:G442–7. 10.1152/ajpgi.00190.2017 28774870 PMC5792209

[11] Meyer-Gerspach AC Wölnerhanssen B Beglinger B Nessenius F Napitupulu M Schulte FH. Gastric and intestinal satiation in obese and normal weight healthy people. Physiol Behav. (2014) 129:265–71. 10.1016/j.physbeh.2014.02.043 24582673

[12] Davis J Camilleri M Eckert D Burton D Joyner M Acosta A. Physical activity is associated with accelerated gastric emptying and increased ghrelin in obesity. Neurogastroenterol Motil. (2020) 32:e13879. 10.1111/nmo.13879 32390274 PMC7606341

[13] Carrio I Estorch M Serra-Grima R Ginjaume M Notivol R Calabuig R. Gastric emptying in marathon runners. Gut. (1989) 30:152–5. 10.1136/gut.30.2.152 2703139 PMC1378293

[14] Horner KM Byrne NM Cleghorn GJ King NA. Influence of habitual physical activity on gastric emptying in healthy males and relationships with body composition and energy expenditure. Br J Nutr. (2015) 114:489–96. 10.1017/S0007114515002044 26168984

[15] Horner KM Byrne NM Cleghorn GJ Näslund E King NA. The effects of weight loss strategies on gastric emptying and appetite control. Obes Rev. (2011) 12:935–51. 10.1111/j.1467-789X.2011.00901.x 21729233

[16] Levin F Edholm T Schmidt PT Gryback P Jacobsson H Degerblad M. Ghrelin stimulates gastric emptying and hunger in normal-weight humans. J Clin Endocrinol Metab. (2006) 91:3296–302. 10.1210/jc.2005-2638 16772353

[17] Jones KL Russo A Berry MK Stevens JE Wishart JM Horowitz M. A longitudinal study of gastric emptying and upper gastrointestinal symptoms in patients with diabetes mellitus. Am J Med. (2002) 113:449–55. 10.1016/S0002-9343(02)01228-7 12427492

[18] Kaji M Nomura M Tamura Y Ito S. Relationships between insulin resistance, blood glucose levels and gastric motility: an electrogastrography and external ultrasonography study. J Med Invest. (2007) 54:168–76. 10.2152/jmi.54.168 17380029

[19] Boulé NG Weisnagel SJ Lakka TA Tremblay A Bergman RN Rankinen T. Effects of exercise training on glucose homeostasis: the HERITAGE family study. Diab Care. (2005) 28:108–14. 10.2337/diacare.28.1.108 15616242

[20] Chanoine JP Mackelvie JK Barr IS Wong KAC Meneilly SG Elahi DH. GLP-1 and appetite responses to a meal in lean and overweight adolescents following exercise. Obesity. (2008) 16:202–4. 10.1038/oby.2007.39 18223636

[21] Mackelvie KJ Meneilly GS Elahi D Wong AC Barr SI Chanoine JP. Regulation of appetite in lean and obese adolescents after exercise: role of acylated and desacyl ghrelin. J Clin Endocrinol Metab. (2007) 92:648–54. 10.1210/jc.2006-1028 17119003

[22] Hagobian T Sharoff C Braun B. Effects of short-term exercise and energy surplus on hormones related to regulation of energy balance. Metabolism. (2008) 57:393–8. 10.1016/j.metabol.2007.10.016 18249213

[23] Whyte LJ Gill JMR Cathcart AJ. Effect of 2 weeks of sprint interval training on health-related outcomes in sedentary overweight/obese men. Metabolism. (2010) 59:1421–8. 10.1016/j.metabol.2010.01.002 20153487

[24] Colley R Hills A King N Byrne N. Exercise-induced energy expenditure: Implications for exercise prescription and obesity. Patient Educ Couns. (2010) 79:327–32. 10.1016/j.pec.2010.03.001 20392589

[25] Manthou E Gill J Wright A Malkova D. Behavioural compensatory adjustments to exercise training in overweight women. Med Sci Sports Exerc. (2010) 42:1121–8. 10.1249/MSS.0b013e3181c524b7 19997033

[26] King NA Caudwell P Hopkins M Byrne NM Colley R Hills AP. Metabolic and behavioral compensatory responses to exercise interventions: barriers to weight loss. Obesity. (2007) 15:1373–83. 10.1038/oby.2007.164 17557973

[27] Stubbs R Hughes D Johnstone A Whybrow S Horgan G King N. Rate and extent of compensatory changes in energy intake and expenditure in response to altered exercise and diet composition in humans. Am J Physiol Regul Integr Comp Physiol. (2004) 286:R350–8. 10.1152/ajpregu.00196.2003 14707013

[28] Westerterp K. Pattern and intensity of physical activity. Nature. (2001) 410:539. 10.1038/35069142 11279482

[29] Slentz CA Duscha BD Johnson JL. Effects of the amount of exercise on body weight, body composition, and measures of central obesity: strride - a randomized controlled study. Arch Int Med. (2004) 164:31–9. 10.1001/archinte.164.1.31 14718319

[30] Hunter GR Weinsier RL Bamman MM Larson DE. A role for high intensity exercise on energy balance and weight control. Int J Obes Relat Metab Disord. (1998) 22:489–93. 10.1038/sj.ijo.0800629 9665667

[31] Hickson RC Bomze HA Holloszy JO. Linear increase in aerobic power induced by a strenuous program of endurance exercise. J Appl Physiol. (1977) 42:372. 10.1152/jappl.1977.42.3.372 838658

[32] Horner KM Byrne NM Cleghorn GJ King NA. Reproducibility of gastric emptying in overweight and obese males. Clin Nutr. (2014) 33:684–8. 10.1016/j.clnu.2013.09.002 24074547

[33] Keating SE Machan EA O'Connor HT Gerofi JA Sainsbury A Caterson ID. Continuous exercise but not high intensity interval training improves fat distribution in overweight adults. J Obes. (2014) 2014:834865. 10.1155/2014/834865 24669314 PMC3942093

[34] Alkahtani S King N Hills A Byrne N. Effect of interval training intensity on fat oxidation, blood lactate and the rate of perceived exertion in obese men. SpringerPlus. (2013) 2:2193–1801. 10.1186/2193-1801-2-532 24255835 PMC3824717

[35] Heydari M Freund J Boutcher SH. The effect of high-intensity intermittent exercise on body composition of overweight young males. J Obes. (2012) 2012:480467. 10.1155/2012/480467 22720138 PMC3375095

[36] Borg GA. Psychophysical bases of perceived exertion. Med Sci Sports Exerc. (1982) 14:377–81. 10.1249/00005768-198205000-00012 7154893

[37] Wood RE Hills AP Hunter GR King NA Byrne NM. Vo2max in overweight and obese adults: do they meet the threshold criteria? Med Sci Sports Exerc. (2010) 42:470–7. 10.1249/MSS.0b013e3181b666ad 19952821

[38] Rossiter HB Kowalchuk JM Whipp BJ. A test to establish maximum O2 uptake despite no plateau in the O2 uptake response to ramp incremental exercise. J Appl Physiol. (2006) 100:764–70. 10.1152/japplphysiol.00932.2005 16282428

[39] Gaskill SE Ruby BC Walker AJ Sanchez OA Serfass RC Leon AS. Validity and reliability of combining three methods to determine ventilatory threshold. Med Sci Sports Exerc. (2001) 33:1841–8. 10.1097/00005768-200111000-00007 11689733

[40] Matthews D Hosker J Rudenski A Naylor B Treacher D Turner R. Homeostasis model assessment: insulin resistance and β-cell function from fasting plasma glucose and insulin concentrations in man. Diabetologia. (1985) 28:412–9. 10.1007/BF00280883 3899825

[41] Goris AH Meijer EP Kester A Westerterp KR. Use of a triaxial accelerometer to validate reported food intakes. Am J Clin Nutr. (2001) 73:549–53. 10.1093/ajcn/73.3.549 11237930

[42] Sasaki JE John D Freedson PS. Validation and comparison of ActiGraph activity monitors. J Sci Med Sport. (2011) 14:411–6. 10.1016/j.jsams.2011.04.003 21616714

[43] Peeters G van Gellecum Y Ryde G Farías NA Brown WJ. Is the pain of activity log-books worth the gain in precision when distinguishing wear and non-wear time for tri-axial accelerometers? J Sci Med Sport. (2013) 12:S1440–2440. 10.1016/j.jsams.2012.12.002 23294696

[44] Matthews CE Hagströmer M Pober DM Bowles HR. Best practices for using physical activity monitors in population-based research. Med Sci Sports Exerc. (2012) 44:S68–76. 10.1249/MSS.0b013e3182399e5b 22157777 PMC3543867

[45] Mâsse LC Fuemmeler BF Anderson CB Matthews CE Trost SG Catellier DJ. Accelerometer data reduction: a comparison of four reduction algorithms on select outcome variables. Med Sci Sports Exerc. (2005) 37:S544–54. 10.1249/01.mss.0000185674.09066.8a 16294117

[46] Riou M-È Jomphe-Tremblay S Lamothe G Finlayson GS Blundell JE Décarie-Spain L. Energy compensation following a supervised exercise intervention in women living with overweight/obesity is accompanied by an early and sustained decrease in non-structured physical activity. Front Physiol. (2019) 10:1048. 10.3389/fphys.2019.01048 31507431 PMC6714465

[47] Schubert MM Palumbo E Seay RF Spain KK Clarke HE. Energy compensation after sprint-and high-intensity interval training. PLoS ONE. (2017) 12:e0189590. 10.1371/journal.pone.0189590 29244836 PMC5731706

[48] McNeil J Brenner D Courneya K Friedenreich C. Dose–response effects of aerobic exercise on energy compensation in postmenopausal women: combined results from two randomized controlled trials. Int J Obes. (2017) 41:1196–202. 10.1038/ijo.2017.87 28360432 PMC5550560

[49] Riou MÈ Jomphe-Tremblay S Lamothe G Stacey D Szczotka A Doucet É. Predictors of energy compensation during exercise interventions: a systematic review. Nutrients. (2015) 7:3677–704. 10.3390/nu7053677 25988763 PMC4446773

[50] Thomas D Bouchard C Church T Slentz C Kraus W Redman L. Why do individuals not lose more weight from an exercise intervention at a defined dose? An energy balance analysis. Obes Rev. (2012) 13:835–47. 10.1111/j.1467-789X.2012.01012.x 22681398 PMC3771367

[51] Ghoos YF Maes BD Geypens BJ Mys G Hiele MI Rutgeerts PJ. Measurement of gastric emptying rate of solids by means of a carbon-labeled octanoic acid breath test. Gastroenterol. (1993) 104:1640–7. 10.1016/0016-5085(93)90640-X 8500721

[52] Schommartz B Ziegler D Schadewaldt P. Significance of diagnostic parameters in [13C]octanoic acid gastric emptying breath tests. Isot Environ Health Stud. (1998) 33:135–43. 10.1080/10256019708036341 9854848

[53] Horner KM Byrne NM King NA. Reproducibility of subjective appetite ratings and *ad libitum* test meal energy intake in overweight and obese males. Appetite. (2014) 81:116–22. 10.1016/j.appet.2014.06.025 24953196

[54] Drapeau V King N Hetherington M Doucet E Blundell J Tremblay A. Appetite sensations and satiety quotient: predictors of energy intake and weight loss. Appetite. (2007) 48:159–66. 10.1016/j.appet.2006.08.002 17045700

[55] King JA Deighton K Broom DR Wasse LK Douglas JA Burns SF. Individual variation in hunger, energy intake and ghrelin responses to acute exercise. Med Sci Sports Exerc. (2017) 49:1219–28. 10.1249/MSS.0000000000001220 28511192

[56] Dorling JL Höchsmann C Fearnbach SN Apolzan JW Hsia DS Johannsen N. Initial weight change and long-term changes in weight and compensation during supervised exercise training. Med Sci Sports Exerc. (2021). 10.1249/MSS.0000000000002633. [Epub ahead of print]. 33731664 PMC8282755

[57] Ross R. Does exercise without weight loss improve insulin sensitivity? Diab Care. (2003) 26:944–5. 10.2337/diacare.26.3.944 12610063

[58] King DS Baldus PJ Sharp RL Kesl LD Feltmeyer TL Riddle MS. Time course for exercise-induced alterations in insulin action and glucose tolerance in middle-aged people. J Appl Physiol. (1995) 78:17–22. 7713807 10.1152/jappl.1995.78.1.17

[59] Martins C Kulseng B Rehfeld JF King NA Blundell JE. Impact of chronic exercise on appetite control in overweight and obese individuals. Med Sci Sports Exerc. (2012) 45:805–12. 10.1249/MSS.0b013e31827d1618 23247700

[60] Leidy HJ Gardner JK Frye BR Snook ML Schuchert MK Richard EL. Circulating ghrelin is sensitive to changes in body weight during a diet and exercise program in normal-weight young women. J Clin Endocrinol Metab. (2004) 89:2659–64. 10.1210/jc.2003-031471 15181038

[61] Stubbs R Sepp S Hughes DA Johnstone AM Horgan GW King N. The effect of graded levels of exercise on energy intake and balance in free-living men, consuming their normal diet. Eur J Clin Nutr. (2002) 56:129–40. 10.1038/sj.ejcn.1601295 11857046

[62] Whybrow S Hughes DR Johnston P Horgan AM King G Blundell JE. The effect of an incremental increase in exercise on appetite, eating behaviour and energy balance in lean men and women feeding *ad libitum*. Br J Nutr. (2008) 100:1109–15. 10.1017/S0007114508968240 18377694

[63] Myers A Dalton M Gibbons C Finlayson G Blundell J. Structured, aerobic exercise reduces fat mass and is partially compensated through energy intake but not energy expenditure in women. Physiol Behav. (2019) 199:56–65. 10.1016/j.physbeh.2018.11.005 30414399

[64] Blundell JE Gibbons C Beaulieu K Casanova N Duarte C Finlayson G. The drive to eat in homo sapiens: energy expenditure drives energy intake. Physiol Behav. (2020)219:112846. 10.1016/j.physbeh.2020.112846 32081814

[65] Hopkins M Duarte C Beaulieu K Finlayson G Gibbons C Johnstone AM. Activity energy expenditure is an independent predictor of energy intake in humans. Int J Obes. (2019) 43:1466–74. 10.1038/s41366-018-0308-6 30659256

[66] Chin-Chance C Polonsky KS Schoeller DA. Twenty-four-hour leptin levels respond to cumulative short-term energy imbalance and predict subsequent intake. J Clin Endocrinol Metab. (2000) 85:2685–91. 10.1210/jc.85.8.2685 10946866

[67] Blundell JE King NA. Effects of exercise on appetite control: loose coupling between energy expenditure and energy intake. Int J Obes Relat Metab Disord. (1998) 22:22–9. 9778093

[68] King NA. What processes are involved in the appetite response to moderate increases in exercise-induced energy expenditure? Proc Nutr Soc. (1999) 58:107–13. 10.1079/PNS19990015 10343347

[69] Fisher G Brown AW Bohan Brown MM Alcorn A Noles C Winwood L. High intensity interval- vs moderate intensity- training for improving cardiometabolic health in overweight or obese males: a randomized controlled trial. PLoS ONE. (2015) 10:e0138853. 10.1371/journal.pone.0138853 26489022 PMC4619258

[70] Heydari M Boutcher SH. Rating of perceived exertion after 12 weeks of high-intensity, intermittent sprinting. Percept Mot Skills. (2013) 116:340–51. 10.2466/06.15.29.PMS.116.1.340-351 23829159

[71] Roxburgh BH Nolan PB Weatherwax RM Dalleck LC. Is moderate intensity exercise training combined with high intensity interval training more effective at improving cardiorespiratory fitness than moderate intensity exercise training alone? J Sport Sci Med. (2014) 13:702–7. 25177202 PMC4126312

[72] Mourier As Gautier J-Fß De Kerviler E Bigard AX Villette JM Garnier JP. Mobilization of visceral adipose tissue related to the improvement in insulin sensitivity in response to physical training in NIDDM: effects of branched-chain amino acid supplements. Diab Care. (1997) 20:385–91. 10.2337/diacare.20.3.385 9051392

[73] Skovgaard C Brandt N Pilegaard H Bangsbo J. Combined speed endurance and endurance exercise amplify the exercise-induced PGC-1a and PDK4 mRNA response in trained human muscle. Physiol Rep. (2016) 4:e12864. 10.14814/phy2.12864 27456910 PMC4962071

[74] Washburn RA Lambourne K Szabo AN Herrmann SD Honas JJ Donnelly JE. Does increased prescribed exercise alter non-exercise physical activity/energy expenditure in healthy adults? A systematic review. Clin Obes. (2014) 4:1–20. 10.1111/cob.12040 25425128 PMC5996763

[75] Willis EA Herrmann SD Honas JJ Lee J Donnelly JE Washburn RA. Nonexercise energy expenditure and physical activity in the midwest exercise trial 2. Med Sci Sports Exerc. (2014) 46:2286–94. 10.1249/MSS.0000000000000354 24694746 PMC4182343

[76] Jung ME Bourne JE Little JP. Where does hit fit? An examination of the affective response to high-intensity intervals in comparison to continuous moderate-and continuous vigorous-intensity exercise in the exercise intensity-affect continuum. PLoS ONE. (2014) 9:e114541. 10.1371/journal.pone.0114541 25486273 PMC4259348

[77] Tjonna AE Lee SJ Rognmo O Stolen TO Bye A Haram PM. Aerobic interval training versus continuous moderate exercise as a treatment for the metabolic syndrome: a pilot study. Circulation. (2008) 118:346–54. 10.1161/CIRCULATIONAHA.108.772822 18606913 PMC2777731

