# Supplementary material for: Effect of Combined Interval and Continuous Exercise Training on Gastric Emptying, Appetite, and Adaptive Responses in Men With Overweight and Obesity
Source: Front Nutr. 2021 May 28;8:654902. doi: 10.3389/fnut.2021.654902 (PMC8192796; doi:10.3389/fnut.2021.654902)
Supplement: Supplementary file 1 [file Data_Sheet_1.docx]

**Supplementary Material**

**Tables**

**Supplementary Table 1** Physical activity over 24 hours following an acute bout of continuous or HII exercise during week 4 of the exercise intervention^1^

|  | **Continuous** | **HII** | ***P*-value** |
| --- | --- | --- | --- |
| Physical Activity |  |  |  |
| Total Steps | 6856 ± 2808 | 6880 ± 2355 | 0.98 |
| Total AEE (kcal) | 583 ± 250 | 599 ± 328 | 0.87 |
| Vector Magnitude Counts | 450559 ± 136703 | 476360 ± 215826 | 0.71 |
| Time in activity | |  |  |
| Vigorous (min) | 2 ± 2 | 5 ± 9 | 0.31 |
| Moderate (min) | 45 ± 26 | 44 ± 30 | 0.91 |

Data are means ± SD.

^1^Data refers to *n* =11 who had valid data for 24 hours post both continuous and HII sessions in week 4. HII, high intensity interval; AEE, activity energy expenditure estimated from accelerometery.


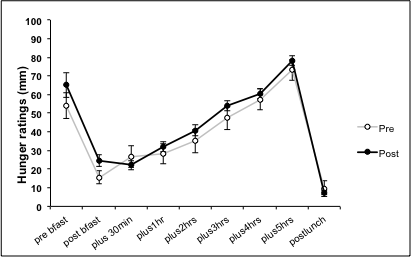
**Figures**

**a)**


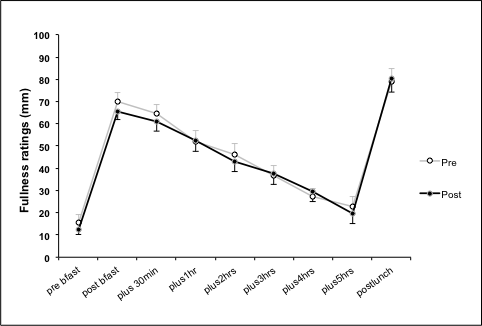


b)


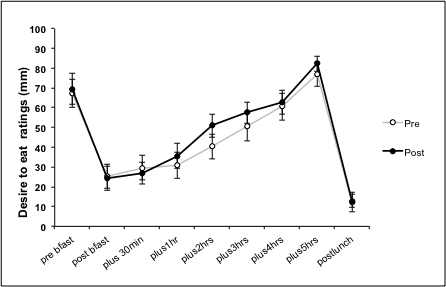
c)

**Supplementary Figure 1** Mean (± SEM) subjective ratings for a) hunger, b) fullness and c) desire to eat over the course of the gastric emptying test morning pre and post the 4-week exercise intervention. n = 15.


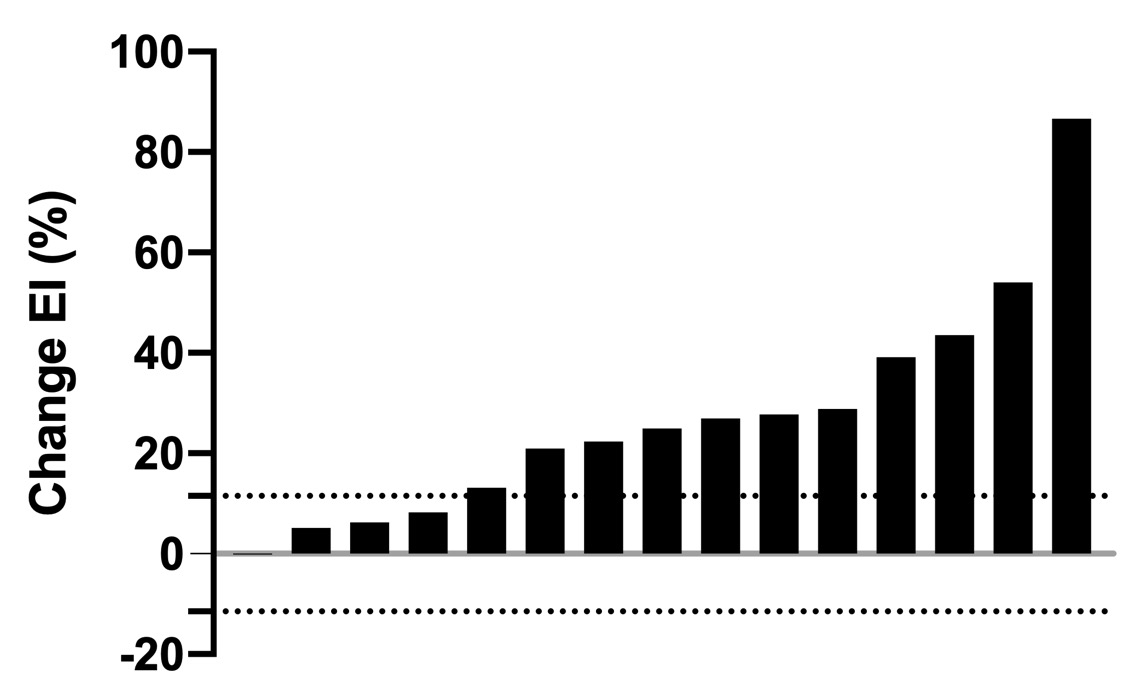


**Supplementary Figure 2.** Individual changes in test meal energy intake (EI) expressed as percentage change from baseline after the 4-week exercise intervention. Each bar represents an individual participant (n=15). Values above zero indicate an increase in EI, values below zero indicate a decrease in EI after the intervention. Dashed horizontal lines represent zones of natural variation in test meal EI (± 12%) based on our previous work (53).


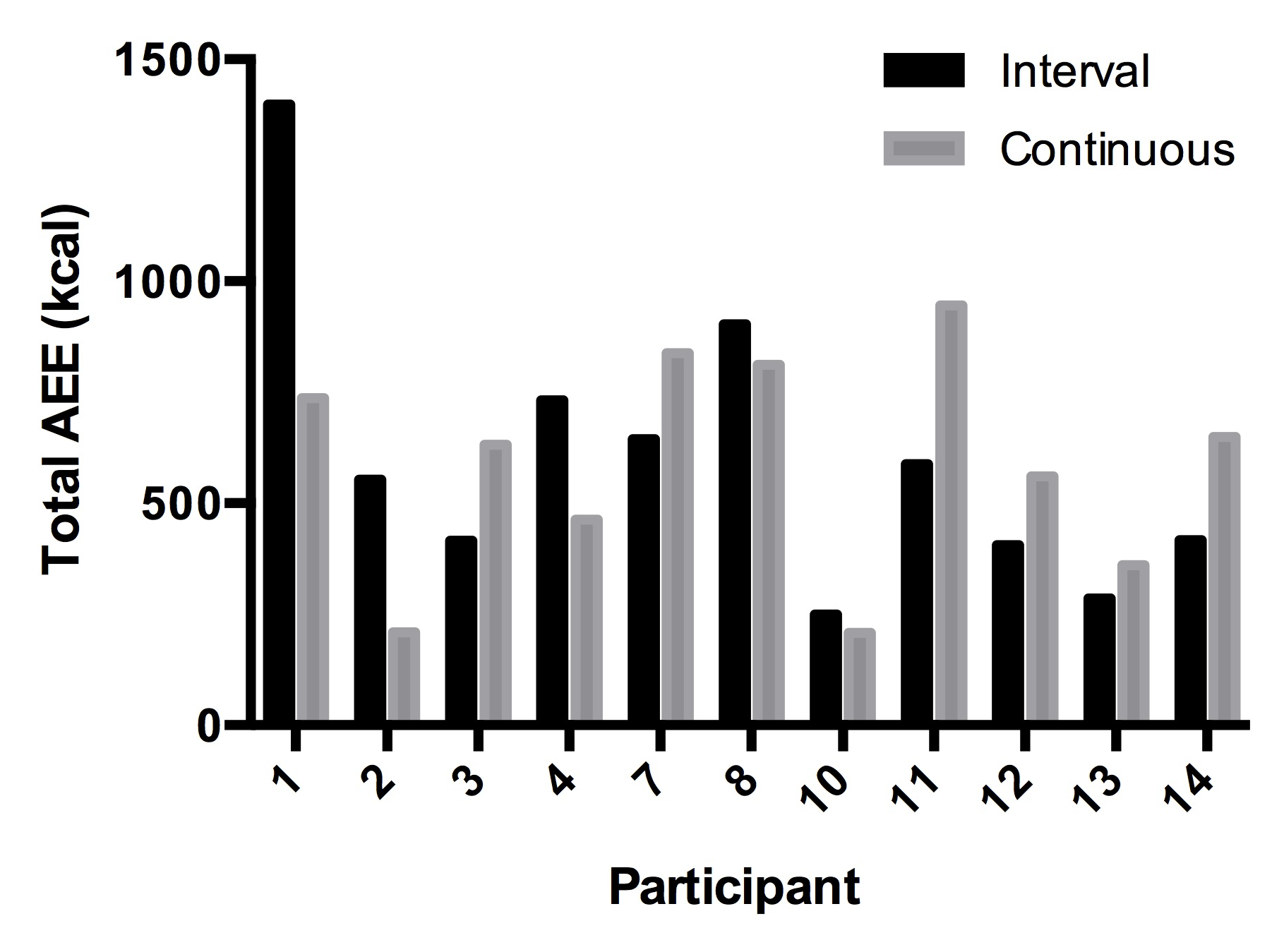


**Supplementary Figure 3.** Individual total estimated AEE (kcal) over 24 hours following interval compared to continuous exercise during week 4 of the intervention, (*n* =11). AEE was estimated from the accelerometer measurement undertaken at baseline and in week 4 of the intervention.
